# Temporal muscle thickness and area with various characteristics data of the elderly patients over 75 with aneurysmal subarachnoid haemorrhage whose World Federation of Neurosurgical Societies grade were I to III

**DOI:** 10.1016/j.dib.2019.104832

**Published:** 2019-11-18

**Authors:** Masahito Katsuki, Yasunaga Yamamoto, Toshiya Uchiyama, Akihiro Nishikawa, Naomichi Wada, Yukinari Kakizawa

**Affiliations:** Department of Neurosurgery, Suwa Red Cross Hospital, Suwa, Nagano, Japan

**Keywords:** Subarachnoid hemorrhage, Elderly, Temporal muscle, Sarcopenia, Prognostic factor

## Abstract

This data informs about the characteristics of elderly patients over 75 with subarachnoid hemorrhage whose World Federation of Neurosurgical Societies (WFNS) grade were I to III. We retrospectively collected data from medical records in our hospital regarding physiological symptoms, laboratory data, radiological data on admission, and outcomes at discharge. Our article entitled “Clinical characteristics of aneurysmal subarachnoid hemorrhage in the elderly over 75; would temporal muscle be a potential prognostic factor as an indicator of sarcopenia? [1]” was based on this data. Remarkably, this is the first dataset on temporal muscle thickness/area and other characteristics of elderly patients over 75 with subarachnoid hemorrhage whose WFNS grade were I to III. Temporal muscle thickness and area were large in the elderly patients with SAH whose outcome were favorable.

**Table:** Specifications Table

Subject	Clinical Neurology
Specific subject area	Neurosurgery and Gerontology
Type of data	Table
How data were acquired	We investigated the medical records of our hospital. We collected objective data from them, such as patients' comorbidities, prognosis, laboratory data, and physiological symptoms. We also investigated the computed tomography images to obtain information on the temporal muscle.
Data format	Raw
Parameters for data collection	From medical records, we collected objective data like laboratory data. Radiological information, such as temporal muscle profile, could be less objective, so we calculated their averages of the left and right from three determinations of each side by two investigators.
Description of data collection	From the subarachnoid hemorrhage databases of our hospital, we retrospectively retrieved the data from all of the 24 patients with aneurysmal subarachnoid hemorrhage over 75. The patients' World Federation of Neurosurgical Societies grade were I to III, and they underwent aneurysm intervention. We collected data regarding physiological symptoms and laboratory data on admission from medical records. Radiological information, including temporal muscle profile, was gained using computed tomography images. Outcomes and activities of daily living before hospitalization were assessed by the modified Rankin Scale. The outcome was evaluated about four weeks after admission in our acute care hospital.
Data source location	Suwa Red Cross Hospital Suwa, Nagano, Japan, 36.0430059, 138.1068495
Data accessibility	With the article
Related research article	Masahito Katsuki, Yasunaga Yamamoto, Toshiya Uchiyama, Naomichi Wada, Yukinari Kakizawa Clinical Characteristics of Aneurysmal Subarachnoid Hemorrhage in the Elderly Over 75; Would Temporal Muscle Be a Potential Prognostic Factor as an Indicator of Sarcopenia? Clinical Neurology and Neurosurgery 186 (2019) 105535 https://doi.org/10.1016/j.clineuro.2019.105535

**Table:** 

**Value of the Data** • First data of the relationship between temporal muscle and outcome of the elderly patients with subarachnoid haemorrhage. • This data can be used as a reference series for clinical neurologist and neurosurgeon to investigate the characteristics of elderly patients with subarachnoid haemorrhage. • The data of temporal muscle thickness/area and modified Rankin Scale at discharge can be used to compare similar elderly patients in other hospital and to investigate novel prognostic factors.

## Data

The dataset in this article describes the characteristics of elderly patients over 75 with subarachnoid hemorrhage whose World Federation of Neurosurgical Societies grade were I to III. We retrospectively collected data from medical records in our hospital regarding physiological symptoms, laboratory data, radiological data on admission, and outcomes at discharge. Table 1 shows the each variables as candidates of prognostic factors.

**Table 1:** Characteristics of the elderly patients over 75 with SAH whose WFNS grade were I to III.

Patient	Age	Sex	Premorbid mRS	Fisher Group	WFNS Grade	Alb (g/dL)	WBC (/mm^3^)	Lymph (/mm^3^)	T-Cho (mg/dL)	TMT (mm)	TMA (mm^2^)	Height (cm)	Weight (kg)	sBP (mmHg)	TG (mg/dL)	LDL (mg/dL)	BS (mg/dL)	HbA1c (%)	Hydrocephalus	Symptomatic vasospasm	Size (mm)	Antithrombotic drug	Treatment	Location	mRS at discharge
1	76	F	0	3	1	3	162.7	1285.33	221	3.94	109.5	142	40	225	206	106	134	6	–	–	14.16	–	Coiling	ICA	0
2	76	F	0	3	1	3.7	74.4	446.4		5.44	198.5	159	44.3	155					–	–	9.46	+	Coiling	BA	0
3	86	F	2	3	2	4	139.5	837	500	5.785	140	150	45.1	138	59	103	239		–	+	15.83	–	Clipping	ICA	0
4	86	F	0	3	2	4.3	60.2	2690.94	203	4.6	173.5	145	43	110		115	141	5.8	–	–	11.75	+	Clipping	ACoA	0
5	79	F	0	3	2	4.1	68.5	3000.3	177	6.535	273	142	49.5	194	186	107	172	6	–	–	7.33	–	Clipping	ICA	0
6	78	F	0	3	2	4	54.9	3189.69	205	5.09	276	154	48.3	188	72	107			–	–	6.6	–	Clipping	ICA	0
7	84	F	0	3	2	4.5	75.3	783.12	203	3.345	107.795	148	40.5	147	60	121	146	6.2	–	–	3.72	–	Clipping	ACoA	1
8	85	F	0	3	2	4.4	190.4	1999.2	162	4.63	107.815	125.8	36.2	176	94	100	141		–	–	2.44	–	Clipping	ICA	1
9	75	F	0	3	2	3.9	75.4	1711.58	147	5.3	215.5	150	48	145	132	76	150	5.1	–	–	6	–	Clipping	VA	2
10	85	F	3	2	2	3.5	95.4	1039.86	196	3.945	113	148	43.5	152	70	127	132	5.4	–	–	2.7	–	Clipping	ACoA	3
11	83	F	1	3	2	4.8	125.4	514.14	295	5.095	218	155	49.6	147	79		250	6.9	+	–	8.1	–	Clipping	ICA	4
12	78	F	0	3	2	3.7	73.3	1092.17		2.94	156.5	148	41.4	188			126		+	–	6.62	+	Clipping	MCA	4
13	81	F	0	3	2	4.1	75	1192.5	206	4.68	227.5	146	31.4	145	109	102	208	5.7	+	–	9.19	–	Clipping	ICA	4
14	81	F	4	3	2	4	62.2	2892.3	240	2.75	45.5	162	55.8	188	116	165	132		–	–	2.3	–	Coiling	BA	4
15	88	F	0	3	2	4.1	58.8	2357.88	163	2.5	50	153	36.6	177	43	86	127	5.4	–	+	5.1	–	Coiling	BA	4
16	93	F	4	3	2	3.6	84.5	481.65	180	3.77	117.5	150	44.3	132	59	109	121	6.6	–	–	5.4	–	Clipping	ACoA	4
17	96	F	0	4	2	3.5	54.6	589.68		2.63	51.35	140	31.9	133			149	5.6	–	–	8	–	Clipping	ACA	5
18	77	F	0	2	1	4.3	56.1	1789.59	222	5.595	145.935	151	55.8	135	187	132	104	5.7	–	–	5.34	–	Clipping	ACoA	5
19	80	M	0	3	1	3.8	150.1	1065.71	153	3.395	111.5	147.7	46	144	63		170	5.2	–	–	6	–	Clipping	ACoA	0
20	88	M	0	2	1	3.6	73.2	461.16		5.155	274.5	165	47	131	42	71	141	6.1	–	–	2.67	–	Clipping	MCA	0
21	76	M	0	3	2	3.6	115.4	473.14	187	7.505	419.5	176	59.8	157	104	117	417	5.8	–	+	5.26	–	Clipping	MCA	0
22	93	M	1	2	1	3.9	72	792	151	4.6	255.5	149	45.1	153	53				–	–	5.8	–	Clipping	MCA	2
23	85	M	0	2	2	3.7	58.1	772.73	167	5	192	162	48.5	152	117		99	6.4	–	–	3.05	–	Clipping	ICA	3
24	83	M	0	3	2	4.1	135.7	2252.62	277	4.745	178	160	47.4	171	410		288	7.8	+	–	5	–	Clipping	MCA	5

ACA; anterior cerebral artery, ACoA; anterior communicating artery, BA; basilar artery, BP; blood pressure, BS; blood sugar, ICA; internal carotid artery, LDL; low density cholesterol, MCA; middle cerebral artery, mRS; modified Rankin Scale, SAH; subarachnoid hemorrhage, T-Cho; total cholesterol, TG; triglycerides, TMA; temporal muscle area, TMT; temporal muscle thickness, VA; vertebral artery, WBC; white blood cell, WFNS; World Federation of Neurosurgical Societies.

## 2 Experimental design, materials, and methods

From the subarachnoid hemorrhage (SAH) databases of our hospital, we retrospectively retrieved the data from all of the 49 patients with aneurysmal SAH over 75 who had been admitted from 2014 to 2018. Of the 49 patients, 24 patients were described in this report; The patients' World Federation of Neurosurgical Societies grade [2] were I to III, and they underwent aneurysm intervention. We collected data regarding physiological symptoms and laboratory data on admission.

We determined the size of the aneurysm, location of the aneurysm, the temporal muscle thickness (TMT), and temporal muscle area (TMA) based on the results of computed tomography (CT) and computed tomography angiography (CTA) on admission. We used Aquilion ONE (Canon Medical Systems Corporation, Tochigi, Japan) to take CT and CTA images of 0.5 × 0.5 × 1.0 mm voxels. The slice thickness was reconstructed to 5 mm. The window width was adjusted to 300, and the window level was adjusted to 20. The TMT and TMA were measured by two investigators who did not know the patients' outcomes using SYNAPSE V 4.1.5 imaging software (Fujifilm Medical, Tokyo, Japan). The TMT was measured bilaterally perpendicular to the long axis of the temporal muscle at the slice 5 mm above the orbital roof, and calculated using averages of the left and right from three determinations of each side. The TMA was measured manually by tracing the outline of the temporal muscle on the same slice as used for measuring the TMT and computed by the software. We calculated their averages of the left and right from three determinations of each side by two investigators [1].

Symptomatic vasospasm was diagnosed by CT, CTA, magnetic resonance imaging (MRI), or magnetic resonance angiography with symptoms. CT or MRI diagnosed hydrocephalus with symptoms. Outcomes and activities of daily living (ADL) before hospitalization were assessed by the modified Rankin Scale [3] (mRS). We dichotomized ADL before hospitalization and at discharge into favorable (mRS 0–2) or poor (mRS 3–6) about four weeks after admission in our acute care hospital.
